# Case of *Legionella pneumophila* Serogroup 1 Infection Linked to Water Flosser, France

**DOI:** 10.3201/eid3202.251386

**Published:** 2026-02

**Authors:** Céline Slekovec, Adrien Biguenet, Audrey Jeanvoine, Didier Hocquet, Xavier Bertrand

**Affiliations:** Centre Hospitalier Universitaire, Besançon, France (C. Slekovec, A. Biguenet, A. Jeanvoine, D. Hocquet, X. Bertrand); Université Marie et Louis Pasteur, Besançon (C. Slekovec, A. Biguenet, D. Hocquet, X. Bertrand).

**Keywords:** bacteria, legionellosis, Legionella pneumophila, hematology, water flosser, environmental investigation, France

## Abstract

Legionnaires’ disease is a severe respiratory infection that causes increased mortality in hospitalized and immunocompromised patients. We report a nosocomial case in France linked to a water flosser. Our findings underscore the need for vigilance regarding such devices and highlight strategies for their safe management within healthcare settings.

Legionnaires’ disease is a respiratory infection caused by inhaling particles contaminated with *Legionella* spp. bacteria (*1*). Although most cases are community-acquired, mortality is nearly 3 times higher for hospital-acquired infections (8.6% vs. 28.6%) (*2*), especially among immunocompromised patients (*3*). This increase in mortality underscores the need for routine monitoring of water systems and the use of protective measures such as point-of-use filters. Environmental investigations are critical for identifying sources of contamination; however, whereas healthcare facilities have the infrastructure to conduct such investigations, implementation in private homes remains challenging. Of consequence, extra-hospital sources of exposure are likely underrecognized. In this article, we describe a case of nosocomial Legionnaires’ disease traced to a water flosser from the patient’s home.

## The Study

A 48-year-old man with IgG Kappa myeloma was hospitalized in August 2024 for a peripheral autologous hematopoietic stem cell transplantation in the hematology unit of Besançon University Hospital (Besançon, France). On day 4, the patient underwent an autograft without complications. On day 10, a fever developed, and *Escherichia coli* was isolated from blood cultures. We began piperacillin/tazobactam (4 g every 6 hours) therapy adapted to the strain resistance profile, and the following blood cultures were negative. However, the fever persisted, and respiratory symptoms developed on day 16. His condition worsened on day 17, and we transferred the patient to the medical intensive care unit (MICU). At admission in MICU, results of a BinaxNOW Legionella urinary antigen test for *Legionella pneumophila* serogroup 1 (Lp1) (Abbott, https://www.abbott.com) were positive. The same day, we detected Lp1 by using PCR on bronchoalveolar samples and cultured endotracheal aspiration samples. No tests for *Legionella* spp. were conducted before day 17. The respiratory distress syndrome caused by pneumonia led to a stay in MICU with mechanical ventilation, dialysis, and cardiorespiratory arrest without sequelae. The patient experienced multiple organ failure with acute renal failure requiring extrarenal purification. Complications including necrosis of the extremities, sacral pressure ulcer, malnutrition, and amyotrophy developed. His condition gradually improved, enabling his discharge from MICU after 47 days.

Because the incubation period was consistent with acquisition in the hematology unit, we searched for environmental sources of *Legionella* spp. During his hospitalization in the unit, the patient stayed in a room intended for immunocompromised patients, and the sink and shower were equipped with the Filt’Ray 2G anti-*Legionella* filters (Aquatools, https://www.aqua-tools.com). We took water samples from the bathroom in the patient’s room (sink and shower with and without filter and the toilet bowl).

We analyzed samples in accordance with the NF T 90-431 standard (Association Française de Normalization, https://www.afnor.org) for counting *Legionella* spp. We sampled 500 mL of water in sterile vials containing 20 mg sodium thiosulfate. First, we inoculated 0.2 mL of water on glycine, vancomycin, polymyxin, cycloheximide plates (Thermo Fisher Scientific, https://www.thermofisher.com). Then, we filtered 10 mL and 100 mL of water through 0.2-µm pore polycarbonate membranes placed on glycine, vancomycin, polymyxin, cycloheximide media. We incubated the plates at 36°C (+2°C) for 8–11 days. We subcultured suspicious colonies to buffered charcoal yeast extract media with and without cystein and identified them by using matrix-assisted laser desorption/ionization time-of-flight mass spectrometry (Bruker, https://www.bruker.com).

We tested the mixed water from the sink and shower (with the filter and after removing the filter) and the toilet bowl water and did not find evidence of *Legionella* spp. In addition, the 10 routine tests conducted in 2024 on the water network supplying the ward were also all negative for *Legionella* spp. During the environmental investigation, a water flosser (Panasonic, https://www.panasonic.com) was found in the patient’s bathroom. This device was not listed in the inventory performed at admission to the hematology department. The device belonged to the patient, which he used at home with tap water, and it was brought in without informing the healthcare team. Because the patient was unable to be interviewed, we were unable to gather information on the use of the device (i.e., frequency of use during hospitalization, type of water used, frequency of cleaning and disinfection). To sample the water flosser, we filled its tank with sterile water, ran the device, and collected 100 ml of water from the jet. We recovered Lp1 and *L. pneumophila* serogroup 2–14 at a concentration of 300 CFU/L (Figure).

**Figure:**
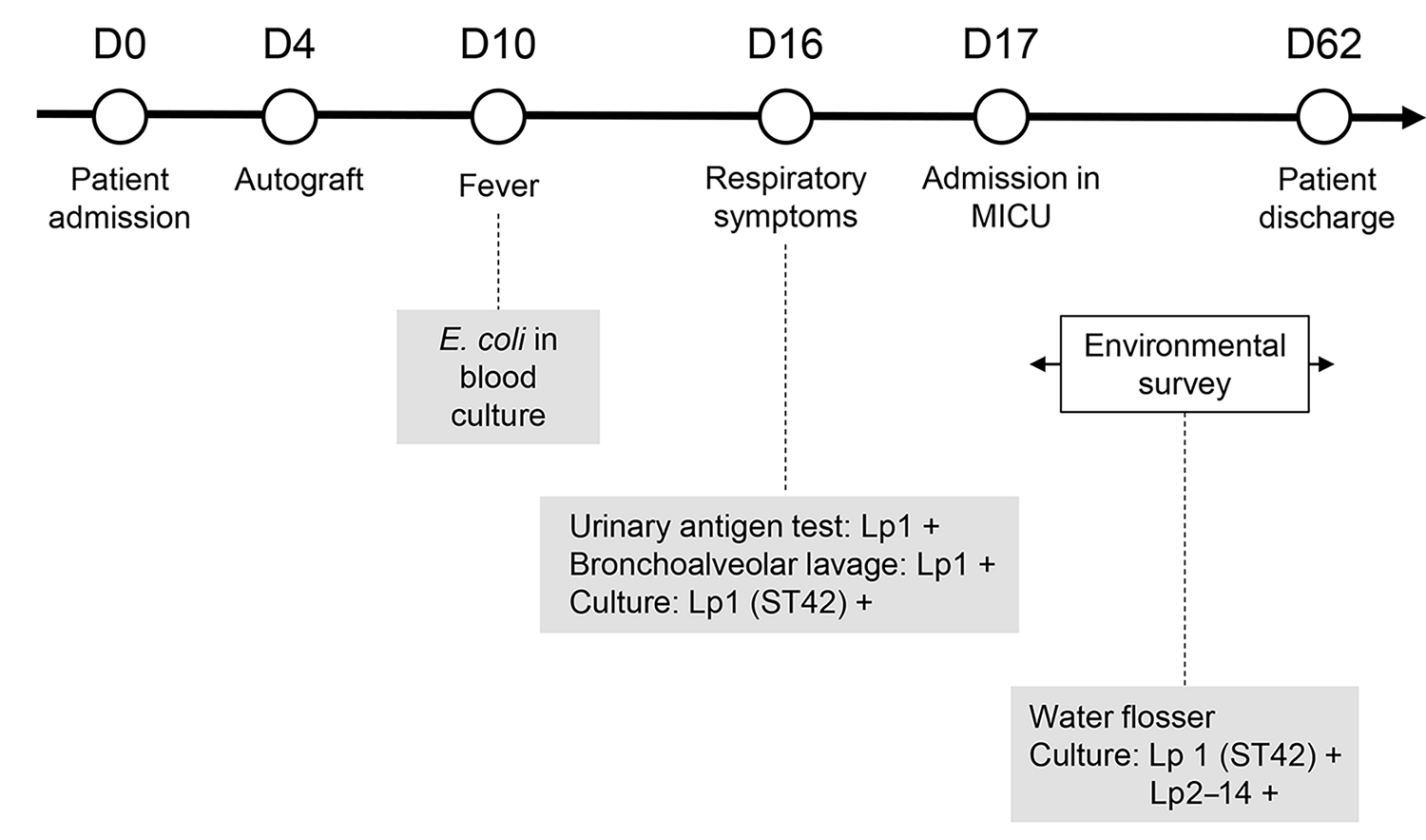
Timeline of patient hospitalization, symptoms, and tests performed in a case of *Legionella pneumophila* serogroup 1 infection linked to water flosser, France. D, day; Lp1, *Legionella pneumophila* serogroup 1; Lp2–14, *Legionella pneumophila* serogroups 2–14; MICU, medical intensive care unit; ST, sequence type; +, positive.

We sequenced the genomes of the 2 Lp1 isolates (1 from the patient and 1 from the water flosser) by using Oxford Nanopore technology (Oxford Nanopore, https://nanoporetech.com). We assembled genomes by using Flye (*4*) and deduced sequence types from whole-genome sequencing data by using legsta (https://github.com/tseemann/legsta). The 2 strains had identical genomes and belonged to sequence type 42, which has been described in France and other countries but is not a major clone (*5*).

Our investigation indicates it is likely the clinical strain and the Lp1 strain identified from the water flosser have a common origin. After this incident, no new cases have been reported in our hospital. The patient was ultimately hospitalized for 62 days, including 47 days in MICU.

## Conclusions

We report a case of Legionnaires’ disease transmitted by a contaminated water flosser. This conclusion was supported by the genomic identity of Lp1 isolates recovered from the patient and water flosser. The other commonly suspected sources, such as shower or sink, or less common, such as toilet (*6*,*7*), were negative for *Legionella* spp. The *Legionella* spp. found in the device (including Lp1, responsible for infection, and *L. pneumophila* serogroup 2–14) likely originated from the patient’s home water network. Unfortunately, no investigation could be conducted to confirm that suspicion. Moreover, the reservoir of the device showed signs of fouling, indicative of the use of nonsterile water and the lack of regular cleaning. Most manufacturers do not advocate for the use of sterile or microbiologically controlled water; instead, they recommend regular cleaning and disinfection procedures. The global water flosser market is experiencing growth ($966.726 million in 2021, projected to reach $1,189 million by 2025), driven by increased awareness of oral hygiene, a growing preference for wireless and portable devices, and guidelines issued by oral health professionals (*8*). Because of the increasing use of such devices, a growing number of immunocompromised patients at risk of Legionnaires’ disease could be exposed. We did not find other cases of contamination through water flossers reported in the literature, indicating a low risk; nevertheless, unrecognized cases cannot be excluded. 

Water flossers might provide favorable conditions for bacterial growth, especially *Legionella* spp. The use of nonsterile water can lead to biofilm accumulation within the device tubing. In addition, the pressurized spray directed into the patient’s mouth may generate aerosols that are readily inhaled. Several studies have demonstrated bacterial colonization of water flossers and the potential transmission of contaminated water jets; in particular, colonization by the major caries-associated pathogen *Streptococcus mutans* appears difficult to prevent (*9*,*10*). Therefore, it is necessary to raise awareness among manufacturers and users regarding the potential risk to immunocompromised patients. In healthcare settings, the use of such devices should be carefully controlled to prevent nosocomial Legionnaires’ disease, and healthcare workers should be aware of the potential risk of devices being brought from the outside into healthcare settings by patients or family members. Our report demonstrates that despite all the measures implemented in hospitals to control the risk for *Legionella* spp. transmission to immunocompromised patients, attention should be paid to unconventional modes of *Legionella* spp. transmission.
